# Age‐related increase of CD38 directs osteoclastogenic potential of monocytic myeloid‐derived suppressor cells through mitochondrial dysfunction in male mice

**DOI:** 10.1111/acel.14298

**Published:** 2024-08-23

**Authors:** Ramkumar Thiyagarajan, Lixia Zhang, Omar D. Glover, Kyu Hwan Kwack, Sara Ahmed, Emma Murray, Nanda Kumar Yellapu, Jonathan Bard, Kenneth L. Seldeen, Spencer R. Rosario, Bruce R. Troen, Keith L. Kirkwood

**Affiliations:** ^1^ Division of Geriatrics, Department of Internal Medicine and Landon Center on Aging University of Kansas School of Medicine Kansas City Kansas USA; ^2^ Research Service Veteran Affairs Kansas City Healthcare System Kansas City Missouri USA; ^3^ Department of Oral Biology, School of Dental Medicine University at Buffalo Buffalo New York USA; ^4^ Department of Oral Microbiology, College of Dentistry Kyung Hee University Seoul Republic of Korea; ^5^ Department of Biostatistics and Data Science University of Kansas Medical Center Kansas City Kansas USA; ^6^ Genomics and Bioinformatics Core, New York State Center of Excellence for Bioinformatics and Life Sciences University at Buffalo Buffalo New York USA; ^7^ Department of Biostatistics and Bioinformatics and Experimental Therapeutics Roswell Park Comprehensive Cancer Center Buffalo New York USA; ^8^ Department of Head and Neck/Plastic and Reconstructive Surgery Roswell Park Comprehensive Cancer Center Buffalo New York USA

**Keywords:** aging, bone resorption, metabolism, myeloid‐derived suppressor cells, osteoclasts

## Abstract

An aged immune system undergoes substantial changes where myelopoiesis dominates within the bone marrow. Monocytic‐MDSCs (M‐MDSCs) have been found to play an important role in osteoclastogenesis and bone resorption. In this study, we sought to provide a more comprehensive understanding of the osteoclastogenic potential of bone marrow M‐MDSCs during normal aging through transcriptomic and metabolic changes. Using young mature and aged mice, detailed immunophenotypic analyses of myeloid cells revealed that the M‐MDSCs were not increased in bone marrow, however M‐MDSCS were significantly expanded in peripheral tissues. Although aged mice exhibited a similar number of M‐MDSCs in bone marrow, these M‐MDSCs had significantly higher osteoclastogenic potential and greater demineralization activity. Intriguingly, osteoclast progenitors from aged bone marrow M‐MDSCs exhibited greater mitochondrial respiration rate and glucose metabolism. Further, transcriptomic analyses revealed the upregulation of mitochondrial oxidative phosphorylation and glucose metabolism genes. Interestingly, there was 8‐fold increase in *Cd38* mRNA gene expression, consistent with the Mouse Aging Cell Atlas transcriptomic database, and confirmed by qRT‐PCR. CD38 regulates NAD^+^ availability, and 78c, a small molecule inhibitor of CD38, reduced the mitochondrial oxygen consumption rate and glucose metabolism and inhibited the osteoclastogenic potential of aged mice bone marrow‐derived M‐MDSCs. These results indicate that the age‐related increase in *Cd38* expression in M‐MDSCs bias the transcriptome of M‐MDSCs towards osteoclastogenesis. This enhanced understanding of the mechanistic underpinnings of M‐MDSCs and their osteoclastogenesis during aging could lead to new therapeutic approaches for age‐related bone loss and promote healthy aging.

## Graphical abstract

Aged M‐MDSCs have increased osteoclastogenesis potential. Transcriptomic analyses revealed the upregulation of mitochondrial oxidative phosphorylation and glucose metabolism genes, especially CD38 or NADase. A specific CD38 inhibitor reduced the mitochondrial oxygen consumption rate and glucose metabolism and inhibited the osteoclastogenic potential of aged mice bone marrow‐derived M‐MDSCs.

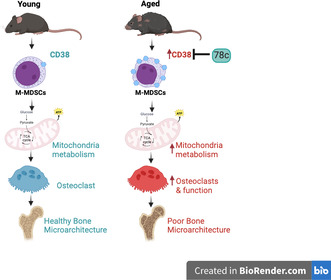

**Abbreviations**
BM: bone marrow
BV/TV: bone volume fraction
CCL2: monocyte chemoattractant protein 1
CCR2: C‐C motif chemokine receptor 2
CD: cluster of differentiation
Ct.Ar: cortical bone area
Ct.Ar/Tt.Ar: cortical area fraction
Ct.Th: cortical thickness
FOV: field of view
GO: gene ontology
IACUC: institutional animal care and use committee
IL: interleukin
kVP: kilovoltage peak
LPS: lipopolysaccharide
Ly6G: lymphocyte antigen 6 complex, locus G6D
Lys6C: lymphocyte antigen 6 complex, locus C1
M‐CSF: macrophage colony‐stimulating factor
MDSC: myeloid‐derived suppressor cells
M‐MDSC: monocytic‐myeloid derived suppressor cells
NAD: nicotinamide adenine dinucleotide
NADH: nicotinamide adenine dinucleotide + hydrogen
OCR: oxygen consumption rate
OXPHOS: oxidative phosphorylation
PMN‐MDSC: polymorphonuclear‐myeloid‐derived suppressor cells
qRT‐PCR: quantitative reverse transcription polymerase chain reaction
RANKL: receptor activator of NF‐kB ligand
ROS: reactive oxygen species
Tb.N: trabecular number
Tb.Sp: trabecular separation
Tb.Th: trabecular thickness
TNF: tumor necrosis factor
TRAP: tartrate‐resistant acid phosphatase
Tt.Ar: total cross‐sectional area inside the periosteal envelope
UMAP: uniform manifold approximation and projection
μA: microamperes
μCT: micro‐computer tomography

## 1 INTRODUCTION

Age‐associated loss of skeletal integrity brings significant health and economic burden in the United States (Khandelwal & Lane, 2023; Li et al., 2017). As people age, osteoclast resorption surpasses osteoblast bone formation, resulting in the loss of bone microarchitecture and mass thus increasing fracture risk in older adults (Li et al., 2017). Women over 50 have a 50% chance of experiencing a fracture in their lifetime, while men over 50 have a 20% chance (Coughlan & Dockery, 2014). Chronic inflammation, known as “inflammaging”, is associated to this age‐related decline in bone microarchitecture, strength, and quality (Briot et al., 2017; Khandelwal & Lane, 2023; Lencel & Magne, 2011). The immune system undergoes dramatic age‐related changes, which continuously progress towards a state of immunosenescence, resulting in changes in the bone marrow that increase the production of many proinflammatory cytokines that further suppress the immune system and promote inflammation (Fulop et al., 2017; Teissier et al., 2022). Immunosenescence within the bone marrow causes a gradual replacement of the different cellular components, including adipocytes, with increased myelopoiesis (Beerman et al., 2010).

One subset of myeloid cells that are particularly expanded during aging by this process are myeloid‐derived suppressor cells (MDSCs), which can contribute to bone loss by differentiating into osteoclasts (Kirkwood et al., 2018; Salminen et al., 2019). This heterogeneous group of immature myeloid cells exhibit immunosuppressive function and expand under chronic inflammatory conditions, including cancer and aging (Jackaman & Nelson, 2014; Pawelec et al., 2021). MDSCs in mice are characterized as CD11b^+^ cellular populations with two distinctive subsets: monocytic MDSCs (M‐MDSCs; CD11b^+^Ly6G^−^Ly6C^hi^) and polymorphonuclear MDSCs (PMN‐MDSC; CD11b^+^Ly6G^+^Ly6C^low^) (Bronte et al., 2016; Kwack et al., 2021). Recent evidence from our laboratory confirms that the osteoclastogenic potential of M‐MDSCs is increased during obesity in inflammatory bone disease, including periodontal disease and post‐traumatic osteoarthritis (Kwack, Zhang, Sohn, et al., 2022; Zhang et al., 2021). However, the metabolic changes and molecular mechanisms underlying the osteoclastogenic potential of M‐MDSCs derived from aged mice remain largely unknown.

Energy metabolism is vital in the formation of osteoclasts and the resorption of bones. This process requires the generation of significant amounts of adenosine triphosphate (ATP) through oxidative phosphorylation and glycolysis. Mitochondrial number, size, and function increase during the differentiation of osteoclasts in preparation for the highly energetic task of resorbing bone (Arnett & Orriss, 2018). Mitochondrial electron transport chain complex 1 generates a considerable amount of ROS, and the inhibition of this complex disturbs osteoclastogenesis (Kwak et al., 2010). In addition to mitochondria, glucose metabolism also plays a role in osteoclast differentiation and function (Li et al., 2020). In anaerobic conditions, glucose is converted to lactic acid by lactate dehydrogenase, and the depletion of this enzyme decreases glucose and mitochondrial metabolism and mature osteoclast formation (Ahn et al., 2016).

The enzymes involved in these metabolic pathways require NAD^+^ (nicotinamide adenine dinucleotide) as a cofactor (Kim et al., 2021), which is essential for cellular metabolism and osteoclastic bone resorption (Kim et al., 2021). However, NAD^+^ declines with aging, which is caused in large part by the age‐related accumulation of CD38 in immune cells (Chini et al., 2020). CD38, a member of the ribosyl cyclase family, is an ectoenzyme on diverse immune cells, including B cells, T cells, natural killer cells, and myeloid cells (Chini et al., 2020; Piedra‐Quintero et al., 2020), catalyzes NAD+ to produce cADPR. Monoclonal antibodies against CD38 have been shown to inhibit osteoclastogenesis in multiple myeloma patients (Costa et al., 2017). However, the effect of inhibiting CD38 activity on the metabolism and osteoclast differentiating potential of bone marrow M‐MDSCs in aged mice has not been explored. This study evaluated the role of CD38 on the osteoclastogenic potential of M‐MDSCs isolated from the bone marrow of aged mice.

## 2 METHODS

### 2.1 Mice

All study protocols were approved by the Institutional Animal Care and Use Committees (IACUC) of the University at Buffalo, and all methods were carried out in compliance with the ARRIVE guidelines. The 6‐month‐old and 24‐month‐old male C57BL/6JNIA mice were acquired from the NIA aging mice colony in Charles River Laboratories (Wilmington, MA). The mice were kept in a controlled temperature and environment under a 12‐h light/12‐h dark cycle, and mice were provided ad libitum access to chow.

### 2.2 Bone microarchitecture and bone histology assessment

Tibias of young and aged mice were fixed in 10% phosphate‐buffered formalin at room temperature and stored (48 h and switched to 70% ethanol) and were scanned with Scanco100 μCT scanner (Scanco Medical, Bruettisellen, Switzerland). Briefly, the tibia were scanned in an ethanol medium at 70 kVP, 114 μA, 15.2 mm FOV, 500 projections/180°, 500 ms integration time, and voxel size of 12 μm. Calibrated three‐dimensional images were generated, reconstructed, and analyzed using AnalyzePro software (AnalyzeDirect, Inc., Overland Park, KS). The parameters of trabecular bone were measured within a specific region of interest located 80 slices (960 μm) from the opening of the epiphyseal growth plate. To select a consistent metaphysis, 80 slices were chosen, starting from 120 slices below to the growth plate to calculate the cortical bone parameters. All images were analyzed individually, and the ROI was applied. The following trabecular and cortical parameters were calculated using AnalyzePro software: bone volume fraction (BV/TV, %), trabecular thickness (Tb.Th, mm), trabecular separation (Tb.Sp, mm), trabecular number (Tb.N, 1/mm), cortical bone area (Ct. Ar), the total cross‐sectional area inside the periosteal envelope (Tt.Ar), cortical area fraction (Ct.Ar/Tt.Ar), and average cortical thickness (Ct.Th). Bone histological assessments were conducted based on our recent publication (Zhang et al., 2024). Briefly, after micro‐CT scans, all tibiae were decalcified in 0.5 M EDTA at pH 8.0 for 14 days and then embedded in paraffin. Longitudinal sections of 5 μm thickness were made and stained by tartrate‐resistant acid phosphatase (TRAP). The counterstain was performed with Fast Green and hematoxylin. TRAP‐positive osteoclast number and surface per total bone surface were quantified on endocortical and trabecular bone in a blinded manner. All micro‐CT and bone histomorphometry analyses and parameters were in accordance with the American Society for Bone and Mineral Research guidelines (Bouxsein et al., 2010; Dempster et al., 2013).

### 2.3 Flow cytometry

Bone marrow, spleen, blood, and mesentery lymph node cells were isolated and washed. The cells were counted, and one million were treated with mouse FcR block (BD Biosciences, NJ) to block non‐specific binding. Then, CD11b, and Ly6C antibodies were used to identify M‐MDSCs in BM, spleen, blood, and mLN (Kwack, Zhang, Kramer, et al., 2022). After staining, the cells were washed with FACS buffer and then fixed in PBS containing 1% paraformaldehyde obtained from Sigma Aldrich. The samples were analyzed using the BD LSR II flow cytometer (BD Biosciences, NJ). Samples were read on the BD LSR II flow cytometer (BD Biosciences, NJ) via FACSDiva version 6.1.3 software. Data were analyzed using FlowJo software version 10.0.9 (FlowJo LLC, OR).

### 2.4 M‐MDSCs isolation

Magnetic and flow sorting were performed to isolate monocytic myeloid‐derived suppressor cells from the bone marrow of young and aged mice. For magnetic sorting, the mouse myeloid‐derived suppressor cell isolation kit (Miltenyi Biotec, Germany) and Miltenyi autoMACS pro were used to sort M‐MDSCs from BM. For flow‐sorting M‐MDSCs, the isolated bone marrow cells were incubated with CD11b, Ly6C, and Ly6G antibodies for 30 min and then sorted for CD11b^+^Ly6G^−^Ly6C^hi^ using BD Biosciences FACSAria or Fusion cell sorter.

### 2.5 Osteoclastogenesis and demineralization activity assay

The M‐MDSCs from the bone marrow of young and aged mice were seeded in a 48‐well plate at 2.5 ×10^5^/well and in an Osteo Assay Surface plate (Corning) for osteoclastogenic differentiation and demineralization activity, respectively. The M‐MDSCs were cultured with monocyte colony‐stimulating factor (M‐CSF; 25 ng/mL, R&D Systems, Bio‐Techne) for 4 days, then osteoclast differentiation was induced with M‐CSF (25 ng/mL) and RANKL (50 ng/mL, R&D Systems, BioTechne) for an additional 6 days. To assess the impact of CD38 inhibition on osteoclastogenic potential of MDSCs, the 78c was administered in the presence of MCSF and RANKL from day 1 of the differentiation of M‐MDSCs isolated from aged and young mice. The cell culture medium was changed daily for 4 days or until the osteoclast formation. The tartrate‐resistant acid phosphatase (TRAP) staining was performed to visualize the preosteoclasts and osteoclasts. Cells with three or more nuclei were considered osteoclasts. The number of osteoclasts was enumerated using Image J. These experiments were reproduced independently, and representative data were presented. The number of demineralized lacunae and area in the Osteo Assay plate was measured by Image J analysis software.

### 2.6 Bulk RNA sequencing

Total RNA was obtained from sorted CD11b^+^Ly6G^−^Ly6C^hi^ M‐MDSCs from young and old mice using RNeasy Mini Kit (Qiagen, USA). The whole‐genome sequencing was performed using NovaSeq 6000 System (Illumina Inc, San Diego, CA). The FastQC and FastQ were used to review the sequencing quality and detect potential contamination, respectively. Bioconductor package DESeq2 version 1.32.0. was used to detect the differentially expressed genes.

### 2.7 Gene ontology enrichment analysis

The gene list from differential expression analysis was processed by removing the blank values and applying significant p‐adj (<0.05) filters. The genes with log2fold change >0 were considered upregulated, and <0 were downregulated. This refined gene list was used for Gene ontology (GO) enrichment. To perform GO enrichment analysis, we utilized the ClusterProfiler package in R software (Yu et al., 2012). First, a list of genes of interest was prepared, consisting of gene symbols. The clusterProfiler package was installed and loaded into the R session. Next, the gene list was subjected to GO enrichment analysis using the enrichGO function provided by ClusterProfiler. This function identified GO terms significantly enriched among the genes of interest compared to the background gene set. The enrichGO function employs the hypergeometric test and adjusts for multiple testing using the Benjamini‐Hochberg method. The resulting enriched GO terms were ranked based on their enrichment significance, determined by the adjusted p‐value. The top enriched GO terms were further visualized using bubble plots that summarize the enriched terms and their associated gene counts.

### 2.8 Tabula Muris Senis dataset analysis

A reference single‐cell transcriptomic atlas for mice called *Tabula Muris Senis* was used to compare the bone marrow myeloid cell distribution and *Cd38* expression in young (3 months) and aged (30 months) mice (“A single‐cell transcriptomic atlas characterizes ageing tissues in the mouse,” 2020). The R package Seurat V4 was used to analyze the droplet scRNAseq data objects and plot the bone marrow immune cell population and myeloid compartment, including promonocytes, monocytes, macrophages, granulopoietic cells, granulocytes, and basophils in young and aged mice. Specifically, the promonocyte populations were analyzed in young and aged mice, and the Cd38 expression was plotted. Briefly, The M‐MDSC module score was generated using the Seurat AddModuleScore function. This function calculates the enrichment of a specific set of genes compared to a set of background control genes. Any values greater than 0 represent enrichment over the background. The M‐MDSC genes used to score were *“Cxcr2”, “S100a9”, “S100a8”, “Ifitm1”, “Lrg1”, “Stfa2l1”, “Retnlg”, “Il1b”, “BC100530”, and “Gm5483”*. Default Seurat parameters were used to create the dimension reduction UMAP (Uniform Manifold Approximation and Projection) projection. The Seurat function AddModuleScore was calculated using genes in GO:0045453 to generate a score for Cd38 and bone resorption for monocyte/granulocyte and promonocytes. Similarly, GO:0009055 genes were created for *Cd38* expression and mitochondrial electron transfer activity in monocyte/granulocyte and for promonocytes (MDSCs).

### 2.9 Metabolic pipeline analysis

The metabolic pipeline utilizes Differentially Expressed Gene output. Scores for each gene were produced by multiplying the −log (adjusted *p*‐value) *logFC on a gene level. Further, absolute value scores were produced by taking the absolute value of the scores for each gene. Using the previously published pipeline (Rosario et al., 2018), we assessed transcriptional metabolic pathway dysregulation in several datasets.

### 2.10 Modeling with Cytoscape

Pathway maps were generated using Cytoscape software (version 3.8.2) (Killcoyne et al., 2009), specifically the VizMapper functions. Pathway maps were adapted from existing pathway maps in WikiPathways (version 3.3.7) (Slenter et al., 2018). The DESeq2 output for DEG analysis was utilized to direct shading of transcripts within the pathway: red (positive fold change), blue (negative fold change), white (non‐statistically significant), or gray (not measured). This allows for the visualization of both transcripts (triangles), proteins (ellipses), and metabolites (rounded rectangles) in a single pathway.

### 2.11 Seahorse extracellular flux analysis

Magnetically sorted young and aged mice M‐MDSCs (400,000 cells per well) were differentiated in the presence of MCSF and RANKL for 48 h. After 48 h, the culture medium was replaced with Seahorse assay medium (103,575, Agilent Technologies, Santa Clara, CA) containing 10 mM glucose, 1 mM pyruvate, and 2 mM glutamine to measure the mitochondrial oxygen consumption rate. A series of mitochondrial inhibitors, 1 μM oligomycin, 1.5 μM FCCP, and 0.5 μM rotenone and antimycin‐A were sequentially injected to measure basal mitochondrial respiration, ATP‐linked respiration, maximal respiration, and non‐mitochondrial respiration. The extracellular acidification rate was also measured in differentiated M‐MDSCs in the presence of the same substrates (glucose, pyruvate, and glutamine). The Seahorse data were normalized to the total DNA content of each well using CyQUNAT cell proliferation assay (C7026, Thermo Fisher Scientific). In order to measure the impact of CD38 inhibition on the oxygen consumption rate of differentiated M‐MDSCs from young and aged mice, the flow sorted M‐MDSCs were plated at the density of 400,000 cells per well with 0.5 μM 78c or DMSO vehicle was administered from day 1 for 48 h in the presence of MCSF and RANKL. At the end of the run, the cell plate was collected and fixed using ice‐cold methanol. The fixed cells were stained with DAPI, and the cells in the center of the wells were imaged using a Keyence fluorescence microscope and counted using the Keyence hybrid cell counter software. This data were normalized to the number of cells. All the measurements were calculated using Agilent Seahorse XF Technology white paper document or manufacturer protocol.

### 2.12 Statistical analysis

GraphPad Prism version 9.5.0 (GraphPad Software Inc., La Jolly, CA) was used to perform unpaired Student t‐tests, one and two‐way analysis of variance (ANOVA) followed by Tukey's multiple comparisons tests with *p* < 0.05 considered significant.

## 3 RESULTS

### 3.1 M‐MDSCs are expanded in aged peripheral tissues but not in the bone marrow

**FIGURE 1:**
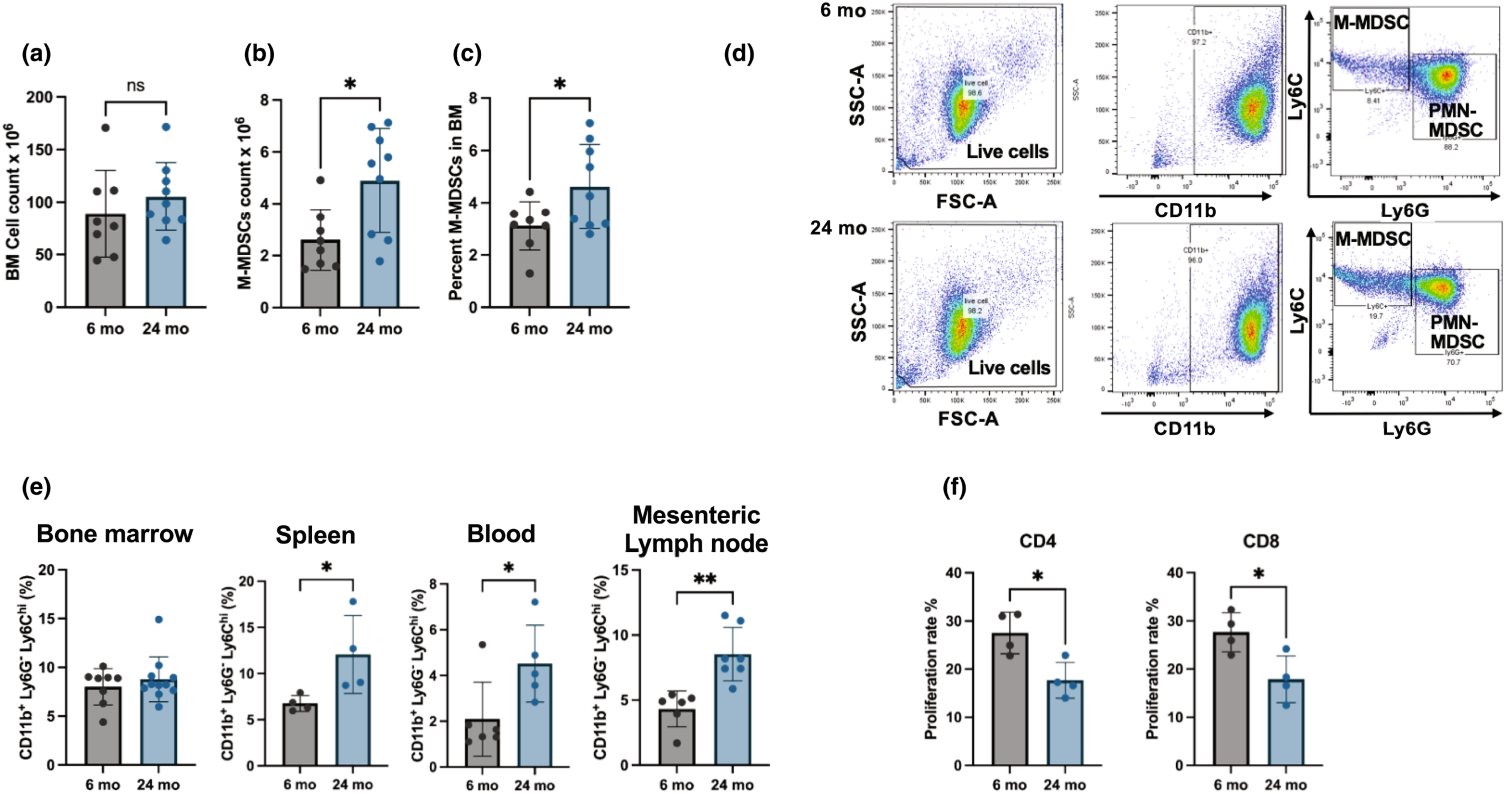
Aged mice myeloid‐derived suppressor cells (MDSCs) expanded in the spleen, blood, and mesentery lymph node but not in BM. Cell count of total bone marrow cells (a), M‐MDSCs after magnetic sorting (b), and percent of sorted M‐MDSCs to the total number of BM cells (c). Flow cytometry gating strategy (d) and analysis of monocytic MDSCs (M‐MDSC; CD11b^+^ Ly6G^−^ Ly6C^hi^) in bone marrow, spleen, blood, and mesenteric lymph nodes (e) from 6‐month‐old young and 24‐month‐old aged male C57BL/6JNIA mice. M‐MDSCs function was tested using T‐cell suppression assay by co‐culturing M‐MDSCs from respective mice and CD4 and CD8 cells from the spleen of young mice (f). Each dot in the dot plots indicates an individual animal. We compared young and aged mice using multiple *t*‐tests. Data are presented as mean ± standard deviation with *p < 0.05, **p < 0.01.

To examine the impact of advanced age on M‐MDSCs, flow cytometer analysis was performed in cells isolated from bone marrow (BM), spleen, blood, and mesentery lymph nodes (mLN). Aged mice BM M‐MDSCs number and percentage M‐MDSCs were increased, despite the total number of BM cells being similar between young and aged mice (Figure 1a–c). The flow cytometry gating strategy was used to calculate the percentage of M‐MDSCs in BM, spleen, blood, and mLN (Figure 1d). The percentage of M‐MDSCs was increased in the spleen, blood, and mLN but not in the BM of aged mice compared to young mice (Figure 1e). The immunosuppressive activity of M‐MDSCs was assessed using a T‐cell suppression assay showing that both young and aged M‐MDSCs reduced the CD4 and CD8 T‐cell proliferation with significantly greater immunosuppressive activity observed in aged M‐MDSCs (Figure 1f). In addition, complete immunophenotyping (PMN‐MDSCs, macrophages, helper T cells, cytotoxic T cells, T helper cells, dendritic cells, and natural killer cells) of BM, spleen, blood, and mLN were performed in young and aged mice (Figure S1a–d).

### 3.2 Aged mice display poor bone microarchitecture and greater osteoclastogenic potential

**FIGURE 2:**
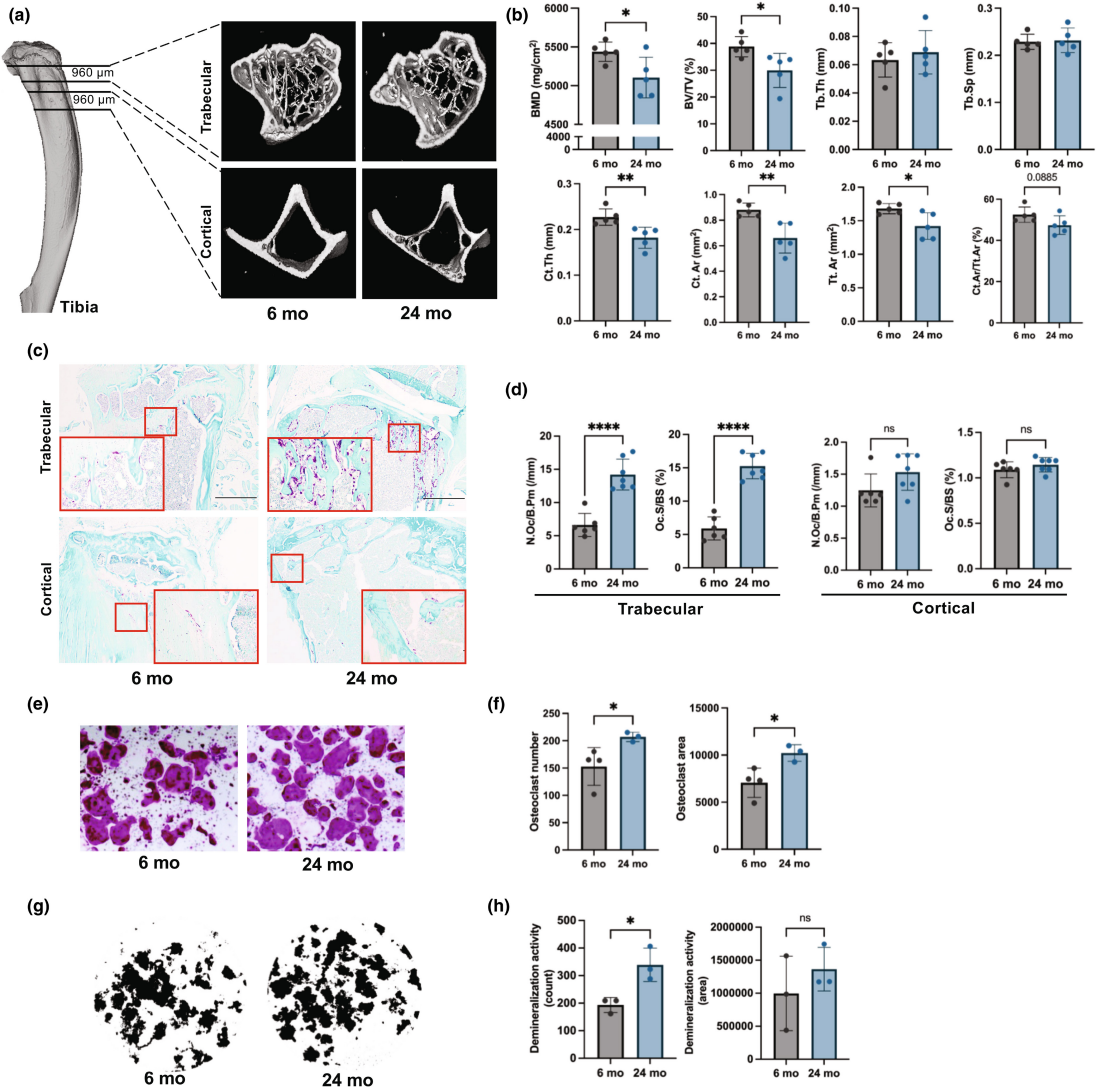
Poor bone microarchitecture in aged mice; the greater osteoclastogenic potential of M‐MDSCs from aged mice. (a) Representative image of the tibia with trabecular and cortical region of interest and micro‐computed tomography (μCT) 3D construction and (b) μCT analysis of tibial trabecular and cortical bone microarchitecture in young (*n* = 5) and aged (*n* = 5) mice. (c) Representative Tartrate‐resistant acid phosphate (TRAP) and fast green counterstained tibia of young (*n* = 6) and aged (*n* = 7) mice. (d) Osteoclast numbers (N.Oc/B.Pm, /mm) and osteoclast surface (Oc.S/BS, %) were calculated on TRAP and fast green counterstained tibia in each group. (e) TRAP‐stained osteoclasts differentiated from M‐MDSCs of young (*n* = 4) and aged (*n* = 3) mice and their total number in a well and area of the osteoclasts (f). Insets are the enlarged area (20×) of smaller red box regions in each 4× image. The scale bar is 500 μm for a 4× image. (g) Representative image of demineralized osteo assay well of young (*n* = 3) and aged (*n* = 3) mice, and (h) the number of demineralization lacunae and demineralized areas of MCSF and RANKL differentiated M‐MDSCs. Each dot in the dot plots indicates an individual animal. We compared young and aged mice using unpaired Student t‐tests. Data are presented as mean ± standard deviation with *p < 0.05, **p < 0.01, ****p < 0.0001.

Bone microarchitecture of the trabecular and cortical region of the tibia was assessed using the Scanco100 micro‐computed tomography system as illustrated (Figure 2a). The aged mice displayed significantly reduced bone mineral density, bone volume fraction (BV/TV), cortical bone area (Ct.Ar), and total cross‐sectional area inside the periosteal envelope (Tt.Ar) compared to young mice (Figure 2b). In addition, the TRAP staining of the tibia and the quantification of a number of osteoclast and osteoclast surfaces revealed an increased number of osteoclasts and osteoclast surface in trabecular bone surface but not in cortical bone surface in aged mice compared to young mice (Figure 2c,d). The bone marrow M‐MDSCs were magnetically sorted from aged and young mice to test their osteoclastogenic potential and osteoclastic demineralization of the synthetic bone surface. The aged mice BM M‐MDSCs exhibited a higher osteoclastogenic capacity in the presence of MCSF and RANKL compared to BM M‐MDSCs from young mice (Figure 2e,f). While the number of osteoclasts increased in aged mice, the variations in their size can impact the overall area covered in the tissue culture plate. Therefore, the osteoclast area was calculated based on the proportion of the total area covered by the osteoclasts in the total well area. This measurement indirectly reflects the size of the osteoclasts. In similar ex vivo culture conditions, the aged mice M‐MDSCs also exhibited elevated osteoclastic activity (Figure 2g,h). The increased demineralization activity of osteoclasts derived from aged M‐MDSCs is likely due to the elevated osteoclastogenic potential of aged M‐MDSCs. Importantly, the flow‐sorted PMN‐MDSCs did not display any osteoclastogenic capacity (Figure S2a,b).

### 3.3 Aged mice M‐MDSCs possess increased mitochondrial oxygen consumption rate and mitochondrial gene expression

**FIGURE 3:**
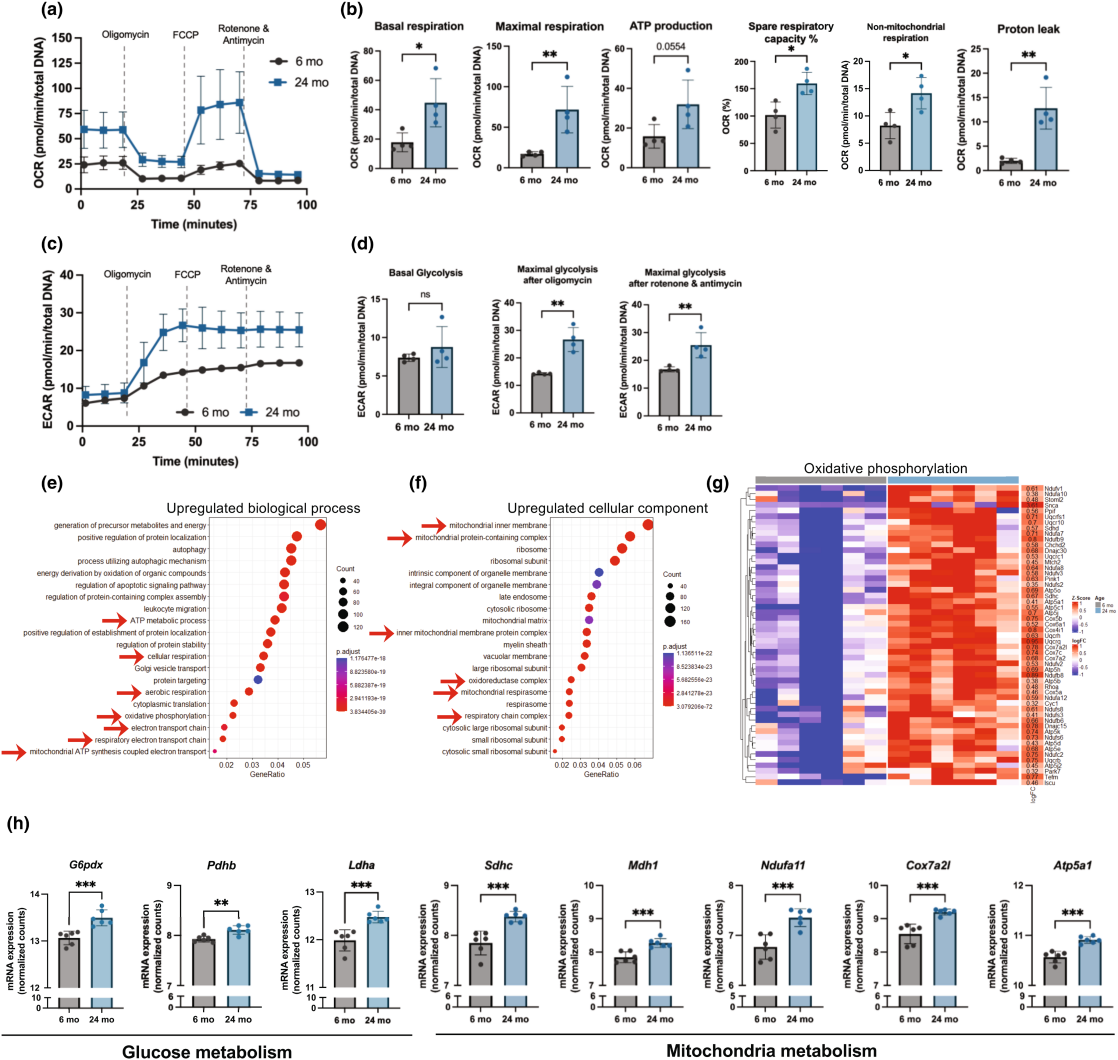
Increased mitochondrial and glucose metabolism in undifferentiated M‐MDSCs and osteoclast progenitors derived from M‐MDSCs of aged mice. A representative mitochondrial oxygen consumption rate (a) and the calculated mitochondrial respiration parameters (b) of young (*n* = 3) and aged (*n* = 4) mice magnetic‐sorted bone marrow M‐MDSCs differentiated for 48 h in the presence of MCSF (macrophage colony‐stimulating factor) and RANKL (Receptor activator of nuclear factor kappa‐Β ligand). A representative extracellular acidification rate (c) and the calculated glycolysis and glycolytic capacity after oligomycin and rotenone and antimycin injections (d). Gene ontology (GO) analysis of the upregulated biological process (e) and cellular component (f) with red arrows indicating mitochondrial metabolism related upregulated biological process and cellular component in aged mice bone marrow M‐MDSCs compared to young M‐MDSCs. (g) Heatmap showing the expression of genes associated with oxidative phosphorylation along with their log2FC change values. (h) Glucose metabolism, mitochondrial Krebs cycle, and electron transport chain genes in young and aged mice bone marrow M‐MDSCs. We compared young and aged mice using unpaired Student t‐tests. Data are presented as mean ± standard deviation with *p < 0.05, **p < 0.01, ***p < 0.001.

To understand the metabolic changes in M‐MDSCs during their differentiation to osteoclasts or osteoclastogenic potential, M‐MDSCs were differentiated in the presence of M‐CSF and RANKL for 48 h, and the measured mitochondrial oxygen consumption rate (OCR) using a Seahorse extracellular flux analyzer (Figure 3a). Differentiated M‐MDSCs from aged mice exhibited increased basal respiration, maximal respiration, spare respiratory capacity, and proton leak (Figure 3b), indicating greater cellular energetics and ability to respond to increased energy demand (spare respiratory capacity). The RANKL‐induced differentiation of aged M‐MDSCs also displayed elevated glycolytic capacity after inhibition of mitochondrial ATP synthase activity and electron transfer function of complexes I and II using oligomycin, and rotenone and antimycin A, respectively (Figure 3c,d). In addition, the glycolysis, glycolytic capacity, and glycolytic reserve were elevated even in the undifferentiated M‐MDSCs harvested from aged mice compared to young mice (Figure S3a,b). To further examine the increased osteoclastogenic potential of aged BM M‐MDSCs, bulk RNA sequencing was performed on M‐MDSCs isolated from the BM of young and aged mice. Gene ontology (GO) enrichment analysis was performed on gene sets to identify the biological processes, cellular components, and molecular functions impacted in M‐MDSCs from aged and young mice. The GO term analysis revealed an upregulated ATP metabolic process, oxidative phosphorylation biological process, and mitochondrial electron transfer‐related cellular components (Figure 3e,f). The list of differentially expressed genes in the oxidative phosphorylation GO term was plotted as a heatmap (Figure 3g). In addition, the aged M‐MDSCs exhibited increased expression of genes related to glucose metabolism (*G6pdx* and *Pdhb*), lactate degradation (*Ldha*), Krebs cycle (*Sdhc* and *Mdh1*), and mitochondrial electron transport chain complexes (*Ndufa11*, *Cox7a2l*, and *Atp5a1*) (Figure 3h).

### 3.4 Aged mice BM M‐MDSCs exhibited greater *Cd38* expression

**FIGURE 4:**
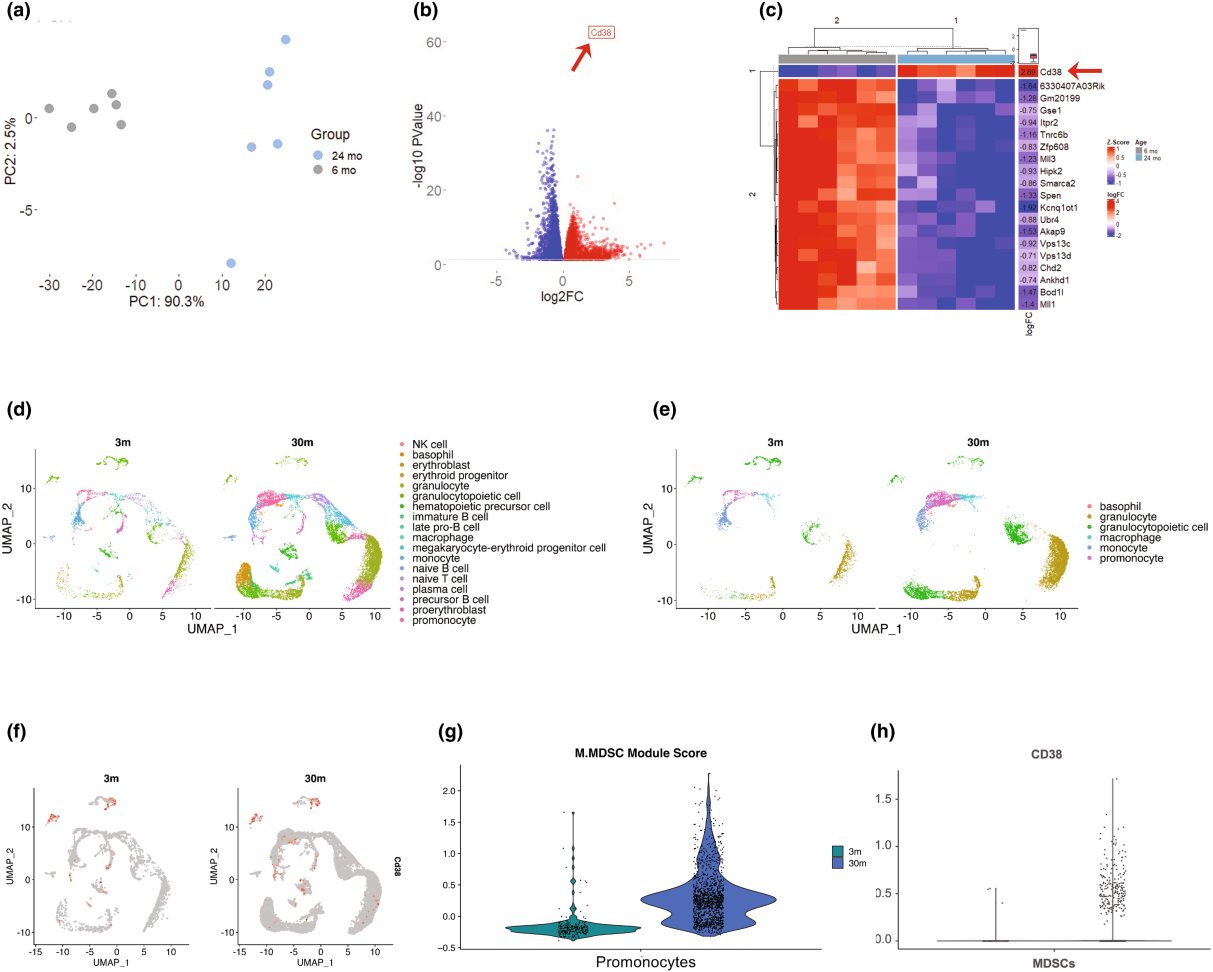
Upregulated *Cd38* expression in aged bone marrow M‐MDSCs. Bulk RNA sequencing of M‐MDSCs from the bone marrow of young (*n* = 6) and aged (*n* = 6) mice displayed distinctive PCA (principal component analysis) plot (a), upregulated (red), and downregulated (blue) differentially expressed genes with highlighted (red arrow and red box) *Cd38* expression (b). Heatmap of top 25 up‐ or down‐regulated genes based on the adjusted p‐value with log2 fold change and average expression value with a red arrow indicating an upregulated *Cd38* gene (c). UMAP (Uniform Manifold Approximation and Projection) plot of 18 different bone marrow immune cells (d) and myeloid compartment, including promonocytes, monocytes, macrophages, granulopoietic cells, granulocytes, and basophils (e) of 3‐month (*n* = 2) and 30‐month‐old (*n* = 3) mice from a droplet single‐cell transcriptomic atlas, *Tabula Muris Senis*. (f) The UMAP plot of *Cd38* expression (red dots) in the myeloid compartment. (g) Violin plot of the M‐MDSC module score in the myeloid compartment. More details are in the methods section. (h) Violin plots of the *Cd38* expression level in the promonocyte or M‐MDSCs population of 3‐month and 30‐month‐old bone marrow single cell transcriptomics atlas.

The initial principal component analysis (PCA) showed a distinctive gene expression profile between aged and young mice BM M‐MDSCs (Figure 4a). Further visual identification of differentially expressed genes with log2 fold changes using Volcano plot revealed the increased and decreased genes in aged M‐MDSCs (Figure 4b), with a marked 8‐fold increase in *Cd38* mRNA expression in aged mice (Figure 4c), which was confirmed by qRT‐PCR (Figure S5a). This indicates that the *Cd38* expression is elevated in BM M‐MDSCs of the aged mice compared to the young mice. *Tabula Muris Senis*, a murine comprehensive single‐cell transcriptomic atlas, was used to confirm whether the expression of *Cd38* mRNA increases with aging. This atlas comprises more than 500,000 cells from 18 organs and tissues across the mouse lifespan (from 1 month to 30 months) (“A single‐cell transcriptomic atlas characterizes ageing tissues in the mouse,” 2020). Transcriptomic signatures of BM from 30‐month versus 3‐month‐old mice were obtained from reference data. As seen in the UMAP, eighteen immune cell subpopulations were identified (Figure 4d), and a noticeable age‐related transcriptomic signature was observed in BM (Figure 4e). The myeloid compartment (6 subpopulations of monocytes and granulocytes) of the 3‐month and 30‐month‐old were analyzed, and identified the age‐related expansion of this subpopulation and concurrent increase in the expression of *Cd38* (Figure 4f). Furthermore, the M‐MDSCs module score was plotted as the violin plot indicated substantial expansion of M‐MDSCs, defined as promonocytes (Figure 4g). The M‐MDSC genes used to score were *Cxcr2, S100a9, S100a8, Ifitm1, Lrg1, Stfa2l1, Retnlg, Il1b, BC100530, Gm5483*. There were more data available for 30‐month‐old mice (*n* = 3; 7324, 3166, and 3006 cells) than for the 3‐month‐old mice (*n* = 2; 1645 and 1845 cells). However, we note there was a fairly high concordance between young and old between the relative proportion of recovered cell types, indicating that the 3‐month samples were likely sequenced at a sufficient depth of sequencing (Figure S4a). The M‐MDSC module score for all the cell populations is displayed in Figure S4b. In addition, in this population, a significant elevation in *Cd38* expression was observed (Figure 4h). Specifically, Figure 4h shows the promonocyte subset, which includes 231 cells for 3‐month and 1333 cells for 30‐month‐old mice. However, the promonocyte population proportionally makes up 6.61% of 3‐month‐old, compared to 9.8% of 30‐month‐olds, indicating that they were relatively close in overall proportion (Figure S4c).

### 3.5 Increased *Cd38* expression is associated with bone resorption

**FIGURE 5:**
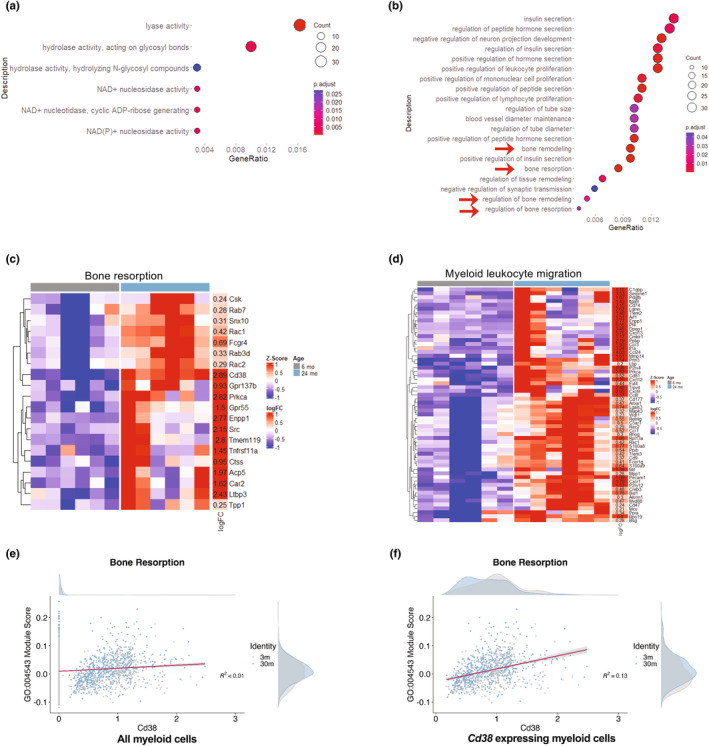
Elevated *Cd38* expression in aged M‐MDSCs associated with bone resorption. *Cd38* gene containing gene ontology (GO) terms of upregulated molecular function (a) and biological process GO terms with green arrows indicating the GO terms related to bone metabolism (b). Heatmap of genes in the bone resorption (c) and myeloid leukocyte migration (d) GO terms with log2 fold change values. UMAP (Uniform Manifold Approximation and Projection) feature plot module score of Cd38 and bone resorption for all myeloid cell populations (e) and *Cd38* expressing myeloid cells (f).

To elucidate the role of the *Cd38* gene on bone resorption in young and aged mice, the *Cd38* gene expressing GO terms were sorted for further analysis. Of those sorted GO terms, genes related to CD38 enzyme activities were upregulated (Figure 5a). In addition, bone resorption, remodeling, and regulation of bone resorption were upregulated (Figure 5b; red arrows). The differentially expressed genes in the bone resorption (Figure 5c) and myeloid leukocyte migration (Figure 5d) were presented as heatmaps. 3‐month and 30‐month‐old mice bone marrow single‐cell transcriptomics data from the *Tabula Muris Senis* dataset (The Tabula Muris Consortium, 2020) were plotted against the *Cd38* and bone resorption GO term GO:0045453 with all cells in the myeloid compartment (Figure 5e), and the myeloid cells expressing *Cd38* (Figure 5f). A module score >0 indicates that the set of genes in the GO‐Term is higher in a specific population than in a random set of background genes. The result indicates a positive trend between the *Cd38‐*expressing myeloid cells and an increase in the expression of genes in the bone resorption GO term (Figure 5f).

### 3.6 Effect of 78c on M‐MDSCs osteoclastogenic potential

**FIGURE 6:**
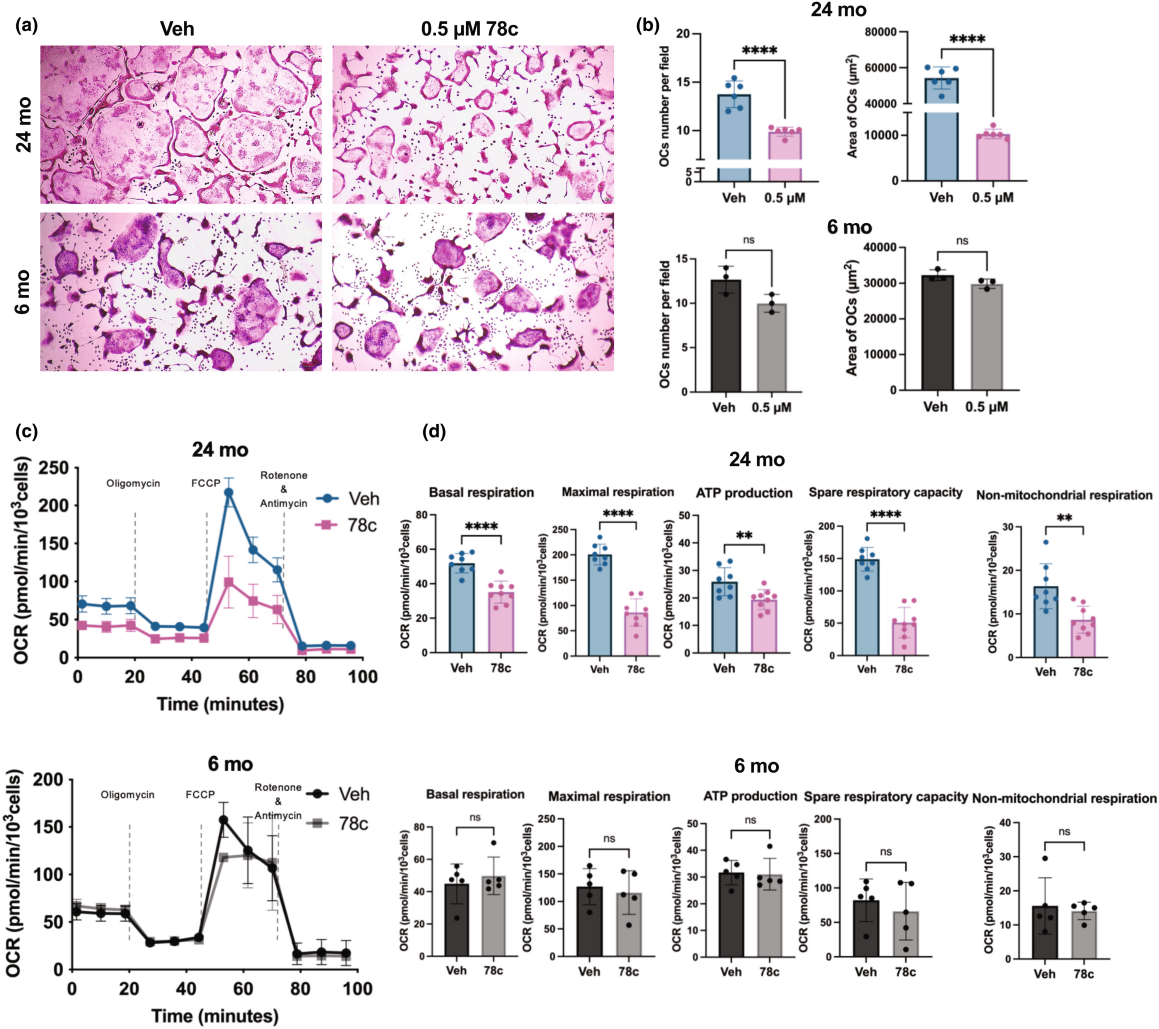
Inhibition of CD38 activity reduced mitochondrial respiration and glycolytic capacity and osteoclast potential of M‐MDSCs from aged mice. TRAP (tartrate‐resistant acid phosphate) staining images (a) and enumeration (b) of osteoclasts differentiated from bone marrow M‐MDSCs from young (*n* = 3; this experiment was reproduced independently, and representative data were presented) and aged (*n* = 6) mice in the presence or absence of 78c. (c) A representative oxygen consumption rate of 48 h differentiated aged (*n* = 3) and young (*n* = 3) mice flow‐sorted bone marrow M‐MDSCs with or without 0.5 μM 78c in the presence of MCSF and RANKL. (d) Basal, maximal, ATP production, spare respiratory capacity, and non‐mitochondrial respiration rate of individual wells after 48 h of vehicle control and 78c treatment. The unpaired Student t‐test was performed to compare the vehicle and treatment groups. Data are presented as mean ± standard deviation with **p < 0.01, ****p < 0.001.

The primary enzyme that breaks down NAD^+^ in the bone marrow is CD38. NAD^+^ and its metabolites play a major role in osteoclast formation and activity. Inhibition of CD38 using a small molecule inhibitor, 78c reduced osteoclast formation or osteoclastogenic potential of aged BM M‐MDSCs, but not the M‐MDSCs from young mice, in the presence of MCSF and RANKL (Figure 6a,b). As preosteoclasts and osteoclasts utilize mitochondria for the resorption function, the mitochondrial oxygen consumption rate of M‐MDSCs‐derived pre‐osteoclasts was tested using Seahorse extracellular flux analysis with or without 0.5 μM 78c. This CD38 inhibitory agent significantly reduced mitochondrial basal, maximal, and non‐mitochondrial respiration, ATP production, and spare respiratory capacity in M‐MDSCs from aged mice but not young mice (Figure 6c,d). Trypan blue dye exclusion assay was performed to determine the cell viability of M‐MDSCs with DMSO or different concentrations of 78c (0.1, 0.5, 1, and 10 μM). No marked cell death was observed in any concentrations compared to the DMSO vehicle control (Figure S5b). CD38 cyclase activity assay confirmed the increase in CD38 cyclase activity in aged mice and the impact inhibitory function of 78c on CD38 enzyme activity. The 0.5 μM 78c reduced the CD38 enzyme function in aged mice but not in young mice bone marrow M‐MDSCs (Figure S5c).

### 3.7 Impact of CD38 on metabolism and RANKL‐driven osteoclastogenesis

**FIGURE 7:**
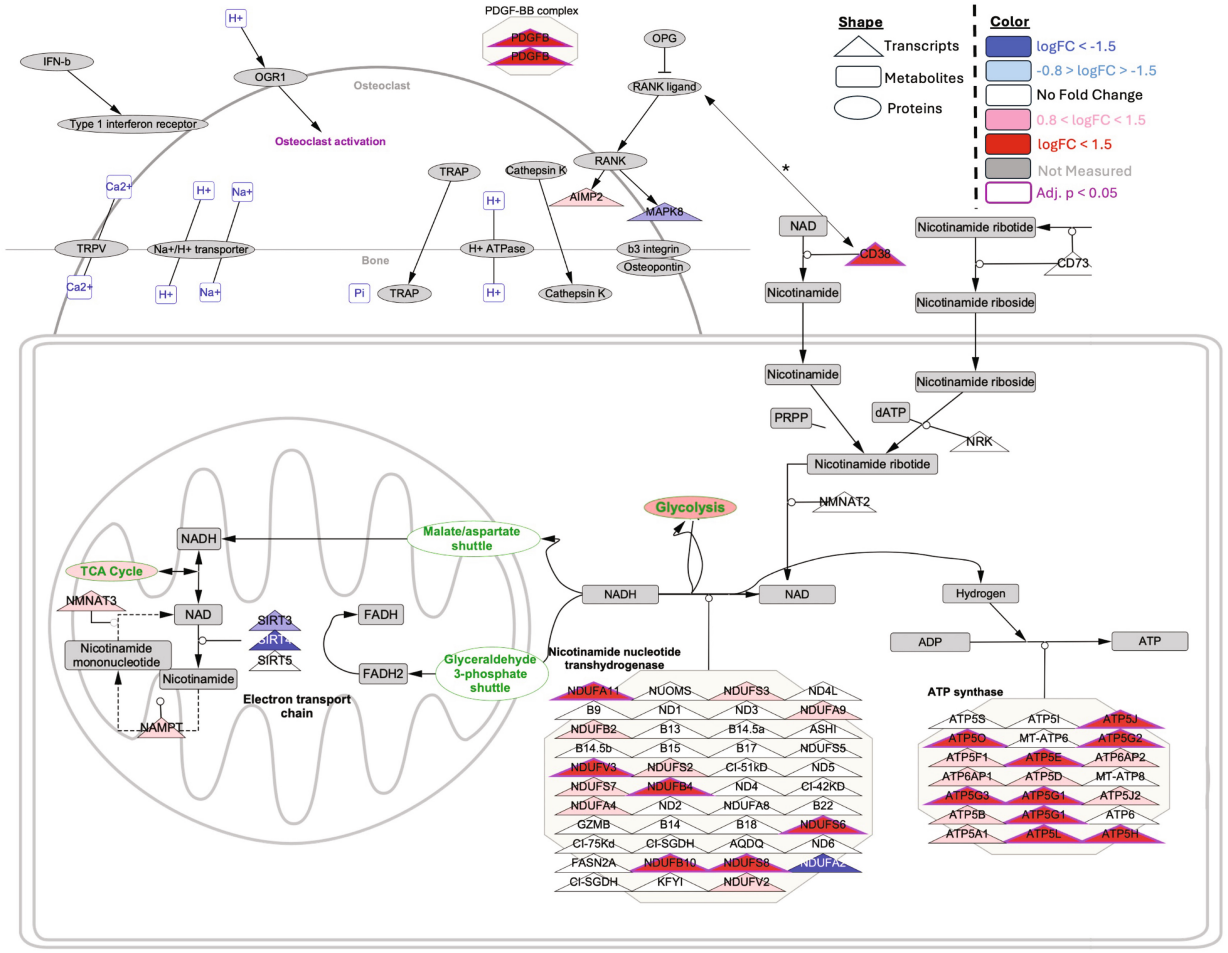
Metabolic pipeline analysis revealed significant transcriptional dysregulation of nicotinamide metabolism, oxidative phosphorylation, glycolysis, and citric acid cycle pathways, amongst others. The complex relationship between RANKL and CD38 (*) is not explored in depth; however, this RANKL‐driven system results in significantly increased CD38 in aged mice, as compared to young mice (red triangle, with purple outline), which, consequently, results in increased transcription of nicotinamide and oxidative phosphorylation, with a large number of transcripts associated with the conversion of NADH to NAD+ and ADP to ATP, especially a large number of ATP5 homologs (red and light red triangles), as exemplified by this transcriptional network.

RNA sequencing was conducted on the bone marrow M‐MDSCs of aged and young mice to detect transcriptional changes and mapped to functional pathways. Metabolic pipeline analysis was used to examine pathways related to nicotinamide metabolism, oxidative phosphorylation, glycolysis, and citric acid cycle, revealing significant transcriptional dysregulation (Figure 7). While the relationship between RANKL and CD38 was not thoroughly explored, it was found that the RANKL‐driven system led to considerably higher levels of CD38 or vice versa in aged mice in contrast to young mice (indicated by a red triangle with a purple outline). This increase in CD38 resulted in amplified transcription of nicotinamide and oxidative phosphorylation, with many transcripts associated with the conversion of NADH to NAD+ and ADP to ATP, particularly a large number of ATP5 homologs indicated by red and light red triangles, as demonstrated in the functional pathway mapped using Cytoscope.

## 4 DISCUSSION

This study shows that M‐MDSCs (CD11b^+^Ly6G^low^Ly6C^hi^) were expanded in the spleen, blood, and mesenteric lymph node but not in the bone marrow of aged mice. The M‐MDSCs from aged mice exhibited elevated *Cd38* gene expression and upregulated differentially expressed genes related to mitochondrial and glucose metabolism. This indicates that aged M‐MDSCs are primed to become osteoclasts compared to M‐MDSCs young mice. In addition, the mitochondrial oxygen consumption and extracellular acidification rates increased in osteoclast progenitors derived from aged M‐MDSCs. Importantly, the targeted inhibition of CD38 using 78c reduced the osteoclastogenesis and mitochondrial and glucose metabolism of osteoclast precursors derived from aged bone marrow M‐MDSCs.

With advancing age, the immune system undergoes dynamic changes characterized by adaptive immunity impairments and low‐grade chronic inflammation activation, which appear to be related to age‐associated chronic inflammatory bone diseases, including osteoporosis, rheumatoid arthritis, and periodontitis (Briot et al., 2017; Coughlan & Dockery, 2014; Khandelwal & Lane, 2023; Lencel & Magne, 2011). This increase in bone loss or fracture incidence that rises with advancing age suggests a pathogenic link between age‐related changes in the immune system and bone loss (Srivastava et al., 2022). As osteoclasts play a crucial role in bone resorption and skeletal maintenance, their function becomes more dominant than osteoblasts during aging. Osteoclasts are multinucleated giant cells derived from the myeloid lineage, which expands during aging (Geiger et al., 2013; Kirkwood et al., 2018; Pawelec et al., 2021) and may increase bone marrow osteoclast precursors, osteoclast formation, and bone resorption (Cao et al., 2005; Kwack, Zhang, Kramer, et al., 2022). About two decades ago, Cao et al. showed the age‐related increased osteoclastogenic potential of myeloid‐lineage bone marrow cells (Cao et al., 2005). Recently, we and others have reported the plasticity of specific myeloid lineage cells, M‐MDSCs, as osteoclast progenitors in metabolic osteoarthritis, obesity‐associated periodontal disease, bone metastasis, and age (Kwack, Zhang, Sohn, et al., 2022; Li et al., 2021; Sawant & Ponnazhagan, 2013; Zhang et al., 2021; Zou et al., 2022). The present study explored the osteoclastogenic potential of bone marrow M‐MDSCs (CD11b^+^Ly6G^−^Ly6C^hi^) from aged mice. The elevated osteoclastogenic potential of aged M‐MDSCs possibly results in increased demineralization activity of osteoclasts derived from this defined population. Previously, other investigators have also demonstrated an age‐related increase in the osteoclastogenic potential of MDSCs (CD11b^+^Ly6C^+^Ly6G^+^), consisting of both M‐MDSCs and PMN‐MDSCs (Z. Li et al., 2021; Zou et al., 2022); however, our lab has noted that the flow‐sorted PMN‐MDSCs (CD11b^+^Ly6G^+^Ly6C^low^) do not efficiently differentiate into osteoclasts.

Osteoclasts require consistent and robust energy substrates to differentiate from osteoclast precursors and bone resorption function (Srivastava et al., 2022). Both mitochondrial respiration and glucose metabolism are required for osteoclastogenesis and activity (Li et al., 2020). Mitochondrial oxidative phosphorylation (OXPHOS) is crucial for the energy‐dependent fusion of monocyte precursors to generate osteoclasts, and poor mitochondrial biogenesis impairs osteoclast differentiation and bone resorption (Li et al., 2020). In support of this, the aged bone marrow M‐MDSCs differentiated to osteoclasts precursors, in the presence of MCSF and RANKL for 48 h, exhibited increased mitochondrial basal and maximal oxygen consumption rate, spare respiratory capacity, proton leak, and non‐mitochondrial respiration. The non‐mitochondrial oxygen consumption rate is an index of oxygen‐consuming processes from other energy sources, such as glucose, for energy‐consuming osteoclastogenesis. Li et al., showed that the *Glut1*‐deficient mouse that spares mitochondrial function in osteoclast progenitors diminishes osteoclast formation (Li et al., 2020). Consistent with these observations, glycolysis or extracellular acidification rate increased in differentiated M‐MDSCs upon inhibition of the mitochondrial electron transport chain complex I and II and mitochondrial ATP production.

CD38 is an enzyme with two distinct activities that degrades NAD+. The first activity is hydrolase, which generates two by‐products, nicotinamide (NAM) and ADP‐ribose (ADPR). The second activity is ADP‐ribosyl cyclase, which generates cyclic‐ADP‐ribose (cADPR), a molecule that plays a role in calcium signaling. CD38 mediates the coupling between calcium sensing and metabolism in osteoclasts, which is essential for bone resorption (Costa et al., 2017). Blocking CD38 with anti‐CD38 mAb inhibits the differentiation of mature osteoclasts from multiple myeloma patients' mononuclear cells (Sun et al., 2003). Additional studies are required to directly address the CD38‐dependent mechanisms that contribute towards enhanced osteoclastogenic activity in the aged skeleton.

As the NAD pool declines with aging, there is a concomitant accumulation of CD38 (NADase) in the immune compartment that contributes towards inflammaging (Camacho‐Pereira et al., 2016). Thus, it is possible that age‐related alteration in the immune system could induce CD38 accumulation in tissues (Chini et al., 2020; Hogan et al., 2019). CD38, an ADP‐ribosyl cyclase/hydrolase that utilizes NAD^+^ as a substrate, is expressed mostly in immune cells (Chini et al., 2020; Hogan et al., 2019). Our findings reveal an approximately eight‐fold increase in *Cd38* gene expression in M‐MDSCs (CD11b^+^Ly6G^−^Ly6C^hi^) isolated from aged bone marrow compared to young controls. Camacho‐Pereira et al. revealed that the knockout of *Cd38* in mice prevented the age‐related decline in cellular NAD level, mitochondrial function, and mitochondrial oxygen consumption rate in liver and spleen cells (Camacho‐Pereira et al., 2016). Interestingly, inhibition of CD38 activity using 78c reduced mitochondrial respiration and glucose metabolism of osteoclast precursors derived from aged bone marrow M‐MDSCs. Consistent with these observations, a CD8^+^ targeted inhibition of CD38 using 78c reversed the defects in mitochondrial dysfunction in a murine lupus model (Chen et al., 2022).

CD38 has long been thought to function as an NAD sensor under conditions of active osteoclast motility and secretion (Sun et al., 2003). Using a globally deficient *Cd38* null mouse, these investigators described a “disordered osteoclast” from 3‐month‐old mutant mice. These data appear contrary to the data presented here, but these differences may be due to the age or type of osteoclast progenitor populations employed. Ongoing efforts have begun to address the role of CD38 in the myeloid compartment of aged mice. In related studies that focused on NAD^+^ supplementation using the NAD^+^ precursor nicotinamide riboside for several months to reverse the aged skeletal phenotype, no differences were observed in osteoclastogenesis but did have major effects on osteoblastogenesis (Kim et al., 2021). Differences from our data may again be due to the osteoclast precursor populations employed or sex, and these studies used only female mice, and the present study used only male mice.

In this study, we used bioinformatic data sets to understand how *Cd38* expression is related to osteoclastic cellular activity. Gene ontology (GO term) analysis of the *Cd38* gene expressing GO terms showed increased bone resorption, remodeling, and regulation of bone resorption GO terms in M‐MDSCs from aged mice. Further analysis of the single‐cell transcriptomic atlas (*Tabula Muris Senis*) also revealed that the *Cd38*‐expressing promonocyte, basophil, and macrophage populations showed a better positive correlation with bone resorption. Therefore, we tested the effect of CD38 inhibition on the osteoclastogenic potential of bone marrow M‐MDSCs from aged mice in the presence of MCSF and RANKL and found that 78c, a small molecule inhibitor of CD38, inhibited the osteoclastogenic potential of bone marrow M‐MDSCs derived from aged mice. Consistently, daratumumab, a monoclonal antibody against CD38, reacted with CD38 expressed on monocytes and inhibited in vitro osteoclastogenesis and bone resorption of mononuclear cells harvested from the bone marrow of multiple myeloma patients (Costa et al., 2017). In addition, an anti‐CD38 antibody inhibited the bone resorption of osteoclasts from young Wistar rats (Sun et al., 1999). In recent years, 78c has been shown to improve metabolic parameters of aging (Tarragó et al., 2018) and health span (Peclat et al., 2022) in mice. Currently, the U.S. Food and Drug Administration approved the monoclonal antibody, daratumumab, against CD38 for managing multiple myeloma, and a few other CD38 blocking antibodies are in clinical trials. Therefore, a deeper understanding of the role of CD38 on early transcriptomic and metabolic changes of M‐MDSCs is essential for pharmacological and/or non‐pharmacological interventions for age‐related bone loss and fracture risk.

Despite the increased *Cd38* expression and osteoclastogenic potential of bone marrow M‐MDSCs in aged mice, there was no increase in the number of M‐MDSCs within the bone marrow of aged mice compared to young control (Flores et al., 2017). This is most likely due to the egress of bone marrow M‐MDSCs into the circulation in response to chronic inflammation during aging. In support of this claim, we found that the M‐MDSC population was expanded in the spleen, blood, and mesentery lymph nodes of aged mice. Indeed, we have made this observation in other conditions where the M‐MDSCs are not expanded in the bone marrow but rather in peripheral tissues. One model system used an RNA binding protein (tristetraprolin or TTP) deficient mouse, where expression of TTP is reduced with aging, and we observed the same type of flow data. In this paper, we went on to define that cell intrinsic differences that led towards M‐MDSC expansion in the peripheral tissues with TTP decline were due to increased regulation of the CCR2‐CCL2 axis (Kwack, Zhang, Kramer, et al., 2022). In addition, we have observed M‐MDSC expansion in peripheral tissues during obesity (Kwack, Zhang, Sohn, et al., 2022) and osteoarthritis (Zhang et al., 2021). In contrast, Li et al. reported an increased accumulation of MDSCs (CD11b^+^Ly6C^hi^Ly6G^+^) in the bone marrow of aged mice (Z. Li et al., 2021). However, there are other studies reported that the aged mice exhibited elevated MDSC (Gr1^+^CD11b^+^) populations, but not the M‐MDSCs (CD11b^+^Ly6G^−^Ly6C^hi^), in the bone marrow, spleen, and lymph nodes compared to young mice (Enioutina et al., 2011; Flores et al., 2017; Jackaman et al., 2013).

The GO term analysis of bulk RNA sequencing of bone marrow M‐MDSCs revealed upregulated OXPHOS‐related biological processes, cellular components, and genes related to glucose and mitochondrial metabolism in aged mice M‐MDSCs compared to young mice, indicating a metabolic shift in undifferentiated aged M‐MDSCs. Further analysis of the GO term of sorted *Cd38* expressing genes showed upregulated bone resorption‐related pathways. In addition, the output of the metabolic pipeline analysis DEG indicated an intricate association between RANKL and CD38. Nevertheless, this system driven by RANKL causes a substantial increase in CD38 in aged mice in comparison to young mice. As a result, there is a rise in the transcription of nicotinamide and oxidative phosphorylation, with numerous transcripts linked to glucose metabolism and the conversion of NADH to NAD+ and ADP to ATP. Collectively, these data indicate that the aged mice M‐MDSCs are metabolically distinctive and primed to differentiate into osteoclasts, and the expression of *Cd38* in M‐MDSCs elevates bone resorption and remodeling. However, further studies are required to investigate the in vivo impact of 78c on the *Cd38* expression on immune cells, especially the M‐MDSC population, the osteoclastogenic potential of MDSCs and their energy metabolism, and bone microarchitecture of aged mice, in a myeloid‐specific CD38 knockout mice to better understand the contribution of the myeloid compartment on bone health during aging. In addition, as aging cells switch metabolism based on their energy demand and alterations in these metabolic phenotypes result in bone pathologies such as osteoporosis and rheumatoid arthritis, future studies should explore the impact of glucose, fatty acid, and amino acid metabolism on the osteoclastogenic potential of M‐MDSCs.

## AUTHOR CONTRIBUTIONS

Keith Kirkwood conceived and designed all the experiments and edited the manuscript. Ramkumar Thiyagarajan and Lixia Zhang performed experiments and wrote the original manuscript draft. Omar Glover, Emma Murray, and Sara Ahmed assisted with osteoclast assays. Kyu Hwan Kwack scanned and analyzed tibia micro‐CT data, provided valuable input in flow cytometry, and edited the manuscript. Nanda Kumar Yellapu, Jonathan Bard, and Spencer Rosario analyzed NextGen RNA sequencing data, single‐cell *Tabula Muris Senis* dataset, and metabolic pipeline utilizing DEG output, respectively. Kenneth Seldeen and Bruce Troen provided valuable scientific insights and edited the manuscript. All authors contributed to the article and approved the submitted version.

## CONFLICT OF INTEREST STATEMENT

The authors have no conflicts of interest.

## Supporting information


Figure S1.

Figure S2.

Figure S3.

Figure S4.

Figure S5.

Data S1.

## Data Availability

The RNA‐seq data in this article is available on NCBI's Gene Expression Omnibus (GEO) through GEO Series accession number GSE 252129.
