# Supplementary figures and images for: Age‐related increase of CD38 directs osteoclastogenic potential of monocytic myeloid‐derived suppressor cells through mitochondrial dysfunction in male mice
Source: Aging Cell. 2024 Aug 23;23(11):e14298. doi: 10.1111/acel.14298 (PMC11561650; doi:10.1111/acel.14298)

**A**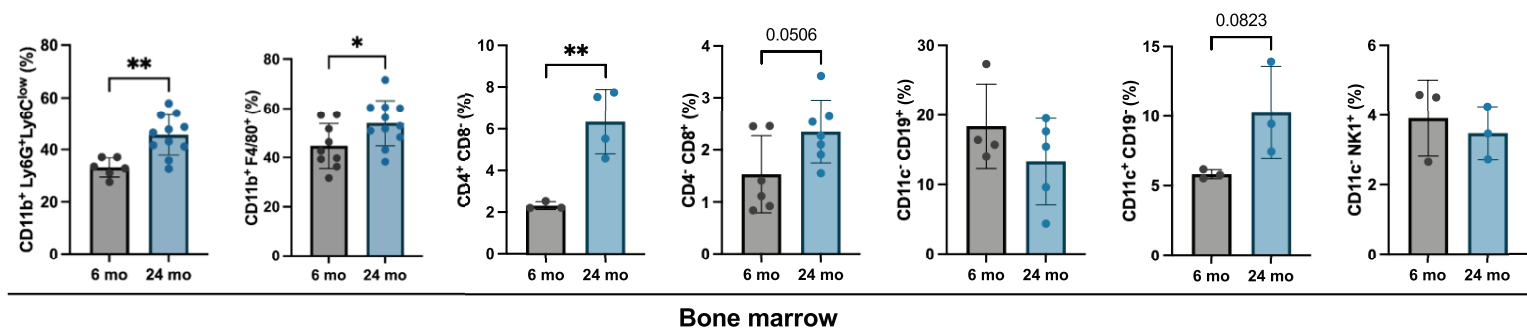**B**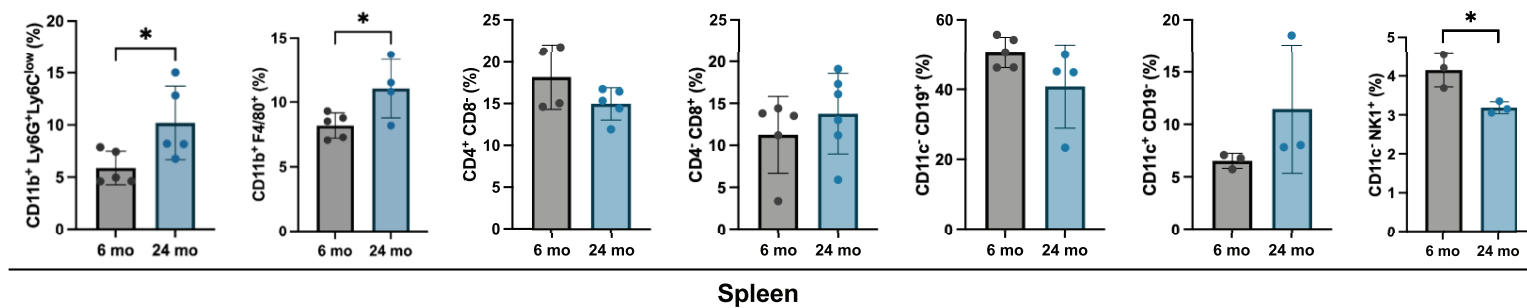**C**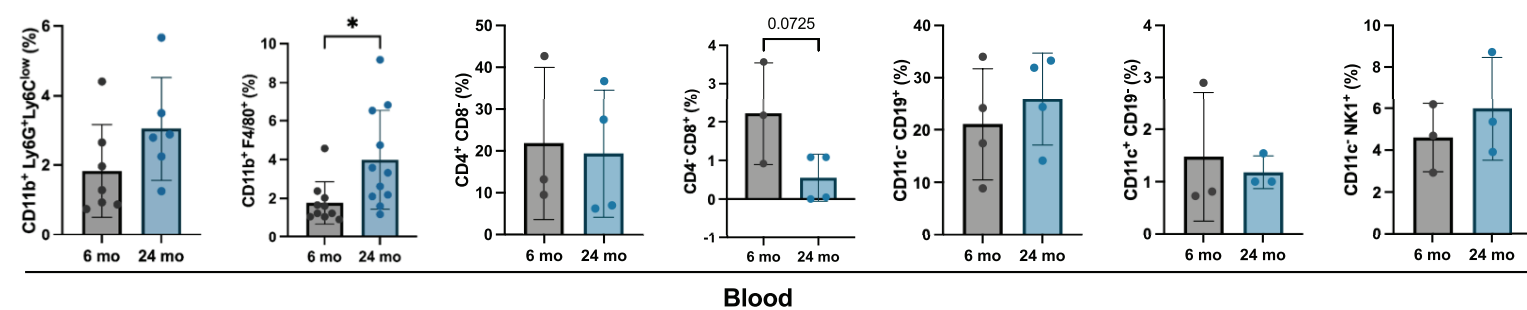**D**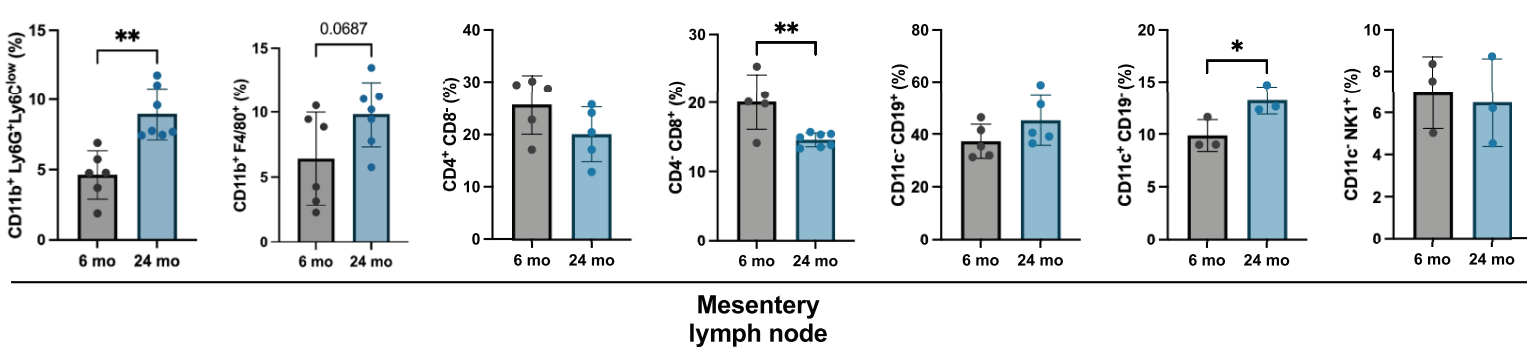

Supplement: Supplementary file 1 — Figure S1. [file ACEL-23-e14298-s006.pdf]

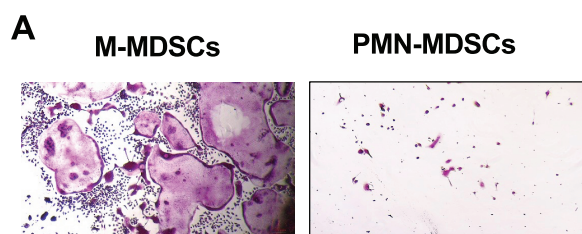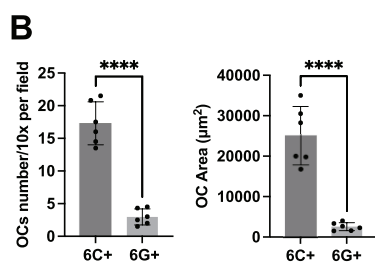

Supplement: Supplementary file 2 — Figure S2. [file ACEL-23-e14298-s001.pdf]

**A**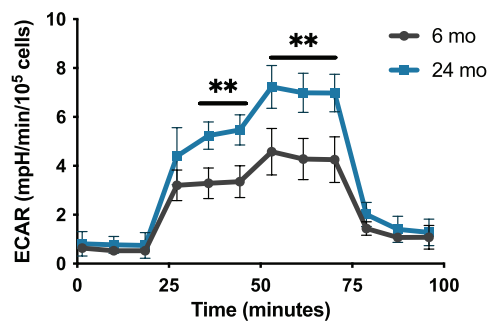**B**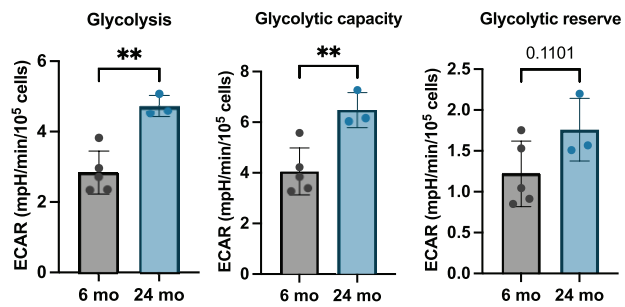

Supplement: Supplementary file 3 — Figure S3. [file ACEL-23-e14298-s004.pdf]

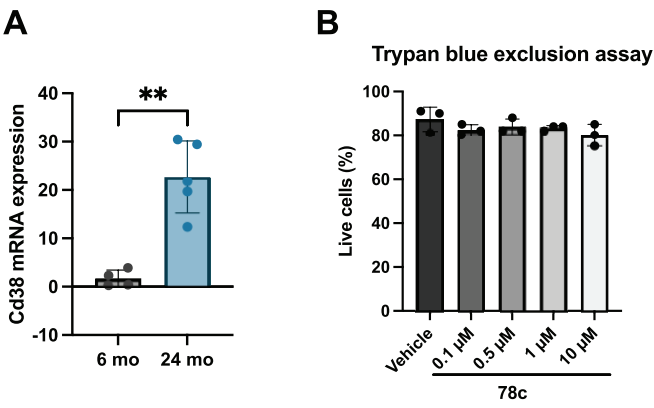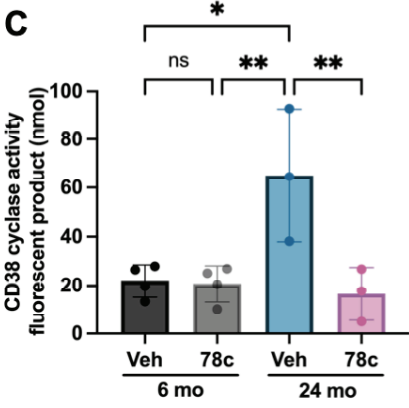

Supplement: Supplementary file 5 — Figure S5. [file ACEL-23-e14298-s005.pdf]
