# Supplementary material for: Age‐related increase of CD38 directs osteoclastogenic potential of monocytic myeloid‐derived suppressor cells through mitochondrial dysfunction in male mice
Source: Aging Cell. 2024 Aug 23;23(11):e14298. doi: 10.1111/acel.14298 (PMC11561650; doi:10.1111/acel.14298)
Supplement: Supplementary file 4 — Figure S4. [file ACEL-23-e14298-s002.pdf]

A

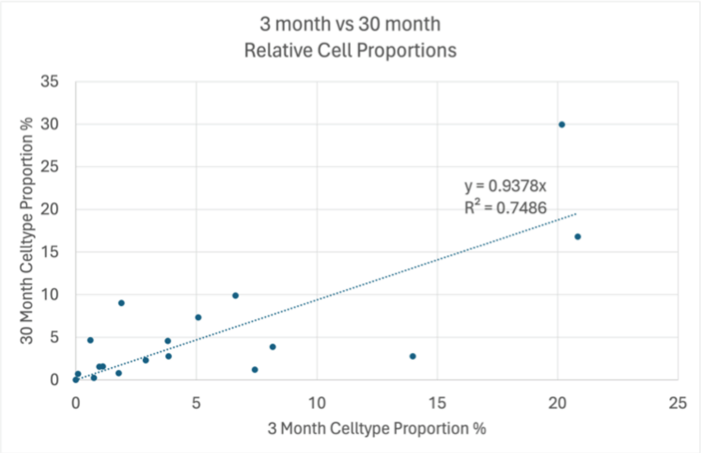

B

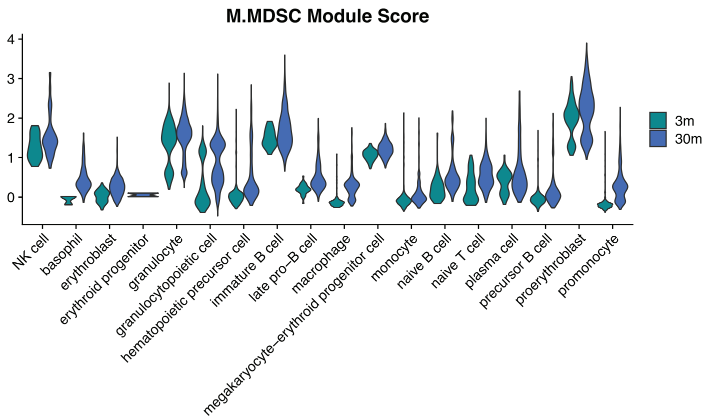

C

Cell Type	3 mo (count)	30 mo (count)	3 mo (%)	30 mo (%)
NK cell	26	31	0.745	0.229
basophil	3	95	0.086	0.704
erythroblast	66	1216	1.891	9.010
erythroid progenitor	0	2	0	0.0148
granulocyte	704	4043	20.172	29.957
granulocytopoietic cell	727	2269	20.831	16.812
hematopoietic precursor cell	285	524	8.166	3.883
immature B cell	21	629	0.6017	4.661
late pro-B cell	34	210	0.9742	1.556
macrophage	101	310	2.894	2.297
megakaryocyte-erythroid progenitor cell	133	616	3.811	4.564
monocyte	488	374	13.983	2.772
naive B cell	62	107	1.776	0.793
naive T cell	134	373	3.839	2.764
plasma cell	39	212	1.117	1.571
precursor B cell	259	163	7.421	1.208
proerythroblast	177	989	5.072	7.328
promonocyte	231	1333	6.619	9.877
