# Supplementary material for: Age‐related increase of CD38 directs osteoclastogenic potential of monocytic myeloid‐derived suppressor cells through mitochondrial dysfunction in male mice
Source: Aging Cell. 2024 Aug 23;23(11):e14298. doi: 10.1111/acel.14298 (PMC11561650; doi:10.1111/acel.14298)
Supplement: Supplementary file 6 — Data S1. [file ACEL-23-e14298-s003.docx]

**Supplementary Methods:**

**Flow cytometry:** Cells isolated from bone marrow, spleen, blood, and mesentery lymph node were stained with cell surface and intracellular markers in three panels. Briefly, Panel 1 included CD11b, Ly6G, and F4/80 antibodies, which helped to identify PMN-MDSCs and macrophages. Panel 2 used CD4, CD25, Foxp3, and CD8 antibodies to identify helper T-cells, regulatory T-cells, and cytotoxic T-cells. Finally, panel 3 was stained with CD11c, CD19, and NK1 antibodies to identify dendritic cells, B cells, and natural killer cells. The BD LSR II flow cytometer was used to analyze the samples. The samples were read via FACSDiva version 6.1.3 software and analyzed using FlowJo software version 10.0.9.

**In vitro osteoclastogenesis assays:** M-MSCSs and PMN-MDSCs were sorted from the bone marrow of young mice and placed in a 48-well plate at a density of 2.5 x 10^5^ cells per well. These cells were then subjected to osteoclastogenic differentiation by adding M-CSF (25 ng/ml) for 3 days and then with the addition of RANKL (50 ng/ml) for an additional 3 days. Tartrate-resistant acid phosphatase (TRAP) staining was performed to visualize the pre-osteoclasts and osteoclasts. Osteoclasts were identified as cells with three or more nuclei. The number and area of osteoclasts generated were quantitated using Image J software.

**Seahorse extracellular flux analysis:** Sorted bone marrow M-MDSCs from young and aged mice were seeded at a density of 400,000 cells per well in an XFe24 well plate coated with CellTak (Corning, NY) to obtain the extracellular acidification rate. 10 mM glucose, 1 µM oligomycin, and 50 mM 2-DG were sequentially injected to measure the glycolysis, glycolytic capacity, and glycolytic reserve of M-MDSCs. The Seahorse data were normalized to a total number of cells. All the measurements were calculated using an Agilent Seahorse XF Technology white paper document or manufacturer protocol.

**Reverse transcription quantitative real-time PCR:** Total RNA was extracted from M-MDSCs using the RNeasy Plus Mini Kit (Qiagen, #74136). Then, 500 ng RNA was used to synthesize complementary DNA (cDNA) using the iScriptTM cDNA synthesis kit (Bio-Rad, #1708890). The cDNA was amplified for quantitative real-time PCR using fast SYBR Green qPCR Master Mix (Applied Biosystems, #4385610) and detected using Bio-Rad CFX Opus 96. The relative mRNA values were calculated using the 2−∆∆CT method. *Gapdh* was used as an internal control in each sample and analyzed for fold change by normalizing it to the mock control. The following primers were used: *Cd38,* forward – GGTCCAAGTGATGCTCAATGGG, reverse - AGCTCCTTCGATGTCGTGCATC and *Gapdh,* forward - GGTTGTCTCCTGCGACTTCA, and reverse - TGGTCCAGGGTTTCTTACTCC.

**CD38 cyclase assay:** Flow-sorted bone marrow M-MSCSs from young and aged mice were seeded in a 24-well plate at a density of 5 x 10^5^ cells per well. The cells were treated with a DMSO vehicle or 0.5 µM 78c in the presence of MCSF and RANKL for 48 hours. After 48 hours, the CD38 cyclase activity was measured using the fluorometric CD38 activity assay kit (ab284540) following the manufacturer’s protocol. The kit utilizes the activity of CD38 to catalyze the conversion of a CD38 substrate to a fluorescent product (Ex/Em = 300/410 nm).

**Trypan exclusion assay:** Young and aged mice bone marrow M-MSCSs were plated in a round bottom 96 well plate at the density of 5 x 10^4^ cells per well. The cells were treated with a DMSO vehicle or 78c (0.1, 0.5, 1, and 10 µM) in the presence of MCSF and RANKL for 24 hours. After 24 hours, the cells were isolated, and the percentage of live cells was counted using Bio-Rad TC20 based on trypan blue presence or absence in a cell. The TC20 auto-detects the presence of trypan blue in the sample to assess cell viability via trypan blue exclusion. Along with the total cell count, the TC20 counter assesses cell viability and provides a live cell count and percentage of live cells.

**Supplementary Figure Legends:**

**Supp. Figure 1:** The flow cytometry analysis of PMN-MDSCs, macrophages, helper T-cells, regulatory T-cells, cytotoxic T-cells, dendritic cells, B cells, and natural killer cells from bone marrow **(A)**, spleen **(B)**, blood **(C)**, and mesentery lymph node **(D)** was conducted using CD11b, Ly6G, F4/80, CD4, CD25, Foxp3, CD8, CD11c, CD19, and NK1 antibodies from 6-month-old young and 24-month-old aged male C57BL/6JNIA mice young mice. Each dot in the dot plot represents an individual mouse. The data were analyzed using multiple t-tests to compare young and aged mice. Data are presented as mean ± standard deviation with *P < 0.05, **P < 0.01.

**Supp. Figure 2:** TRAP staining of flow sorted M-MDSCs and PMN-MDSCs **(A)**, and the number and area of the osteoclasts **(B)** differentiated from M-MDSCs and PMN-MDSCs isolated from bone marrow of young mice (*n*=6). Student t-test was conducted to compare cell populations. Data are presented as mean ± standard deviation with ****P < 0.0001.

**Supp. Figure 3:** A representative extracellular acidification rate **(A)** of young (*n*=3) and aged (*n*=3) mice bone marrow M-MDSCs and the calculated glycolysis, glycolytic capacity, and glycolytic reserve parameters **(B)** after glucose, oligomycin, and rotenone and antimycin injections. Multiple t-tests were conducted to compare young and aged mice. Data are presented as mean ± standard deviation with **P < 0.01.

**Supp. Figure 4:** **(A)** Relative cell proportion of 3-month-old (n=2) and 30-month-old (n=3) mice from Tabula Munis Senis dataset. The M-MDSC module score for all the cell types is presented as a violin plot **(B)** and a Table **(C)** with cell number and proportion of all cell populations.

**Supp. Figure 5:** *Cd38* expression of M-MDSCs isolated from 6-month-old (*n*=4) and 24-month-old (*n*=5) mice, normalized to *Gapdh*, was assessed using reverse transcript quantitative polymerase chain reaction **(A)**. The percent of live trypan blue dye-excluded M-MDSCs treated with different concentrations of 78c (0.1, 0.5, 1, and 10 µM) and vehicle control for 24 hours, *n*=3 **(B)**. **(C)** CD38 cyclase activity of young (*n*=4) and aged (*n*=3) M-MDSCs treated with DMSO vehicle or 0.5 µM 78c for 48 hours. The data were analyzed using multiple t-tests or one-way ANOVA. Data are presented as mean ± standard deviation with *P < 0.05, **P < 0.01.
